# Functional associations of pleuroparenchymal fibroelastosis and emphysema with hypersensitivity pneumonitis

**DOI:** 10.1016/j.rmed.2018.03.031

**Published:** 2018-05

**Authors:** Joseph Jacob, Arlette Odink, Anne Laure Brun, Claudio Macaluso, Angelo de Lauretis, Maria Kokosi, Anand Devaraj, Sujal Desai, Elisabetta Renzoni, Athol U. Wells

**Affiliations:** a Department of Respiratory Medicine, University College London, London, UK; b Centre for Medical Computing, University College London, London, UK; c Department of Radiology, Erasmus MC Rotterdam, The Netherlands; d Imaging Department, Hôpital Cochin, Paris-Descartes University, France; e Department of Respiratory Medicine, Ospedale "Luigi Sacco", University of Milan, Italy; f Interstitial Lung Disease Unit, Royal Brompton Hospital, Royal Brompton and Harefield NHS Foundation Trust, London, UK; g Division of Pneumology, "Guido Salvini" Hospital, Garbagnate Milanese, Italy; h Department of Radiology, Royal Brompton Hospital, Royal Brompton and Harefield NHS Foundation Trust, London, UK

**Keywords:** Hypersensitivity pneumonia, Radiology thoracic, Pleuroparenchymal fibroelastosis, Emphysema, Interstitial lung disease

## Abstract

**BACKGROUND:**

Pleuroparenchymal fibroelastosis (PPFE) has been described in hypersensitivity pneumonitis (HP) yet its functional implications are unclear. Combined pulmonary fibrosis and emphysema (CPFE) has occasionally been described in never-smokers with HP, but epidemiological data regarding its prevalence is sparse. CTs in a large HP cohort were therefore examined to identify the prevalence and effects of PPFE and emphysema.

**Methods:**

233 HP patients had CT extents of interstitial lung disease (ILD) and emphysema quantified to the nearest 5%. Lobar percentage pleural involvement of PPFE was quantified on a 4-point categorical scale: 0 = absent, 1 = affecting <10%, 2 = affecting 10–33%, 3 = affecting >33%. Marked PPFE reflected a total lung score of ≥3/18. Results were evaluated against FVC, DLco and mortality.

**RESULTS:**

Marked PPFE prevalence was 23% whilst 23% of never-smokers had emphysema. Following adjustment for patient age, gender, smoking status, and ILD and emphysema extents, marked PPFE independently linked to reduced baseline FVC (p = 0.0002) and DLco (p = 0.002) and when examined alongside the same covariates, independently linked to worsened survival (p = 0.01).

CPFE in HP demonstrated a characteristic functional profile of artificial lung volume preservation and disproportionate DLco reduction. CPFE did not demonstrate a worsened outcome when compared to HP patients without emphysema beyond that explained by CT extents of ILD and emphysema.

**CONCLUSIONS:**

PPFE is not uncommon in HP, and is independently associated with impaired lung function and increased mortality. Emphysema was identified in 23% of HP never-smokers. CPFE appears not to link to a malignant microvascular phenotype as outcome is explained by ILD and emphysema extents.

## Highlights

• In patients with HP, marked PPFE had a prevalence of 23% in our study cohort.
• 23% of never-smokers with HP demonstrated emphysema on CT imaging.
• Outcome in HP patients with CPFE is explained by CT extents of ILD and emphysema.
• PPFE is independently associated with a worsened outcome in HP.

## Abbreviations

CPFE: combined pulmonary fibrosis and emphysema
CPI: composite physiological index
CT: computed tomography
DLco: diffusing capacity for carbon monoxide
FEV1: forced expiratory volume in the first second
FVC: forced vital capacity
HP: hypersensitivity pneumonitis
ILD: interstitial lung disease
IPF: idiopathic pulmonary fibrosis
Kco: carbon monoxide transfer coefficient
PPFE: pleuroparenchymal fibroelastosis
RA-ILD: rheumatoid arthritis related interstitial lung disease
SD: standard deviation

## 1 Introduction

Over the past fifteen years, the existence of idiopathic pleuroparenchymal fibroelastosis (PPFE) has been increasingly recognised [1,2], and in 2013, PPFE was included in the consensus classification of the idiopathic interstitial pneumonias [3]. However non-idiopathic PPFE has been increasingly reported in association with several interstitial lung diseases (ILD) including idiopathic pulmonary fibrosis (IPF) [2,4,5], hypersensitivity pneumonitis (HP) [2,6] and familial forms of pulmonary fibrosis [1,7]. As yet however, no large-scale CT study has attempted to characterize the prevalence and associations of PPFE occurring in tandem with an ILD.

Several recent studies have also identified the presence of emphysema on CT imaging in never-smoker patients with rheumatoid arthritis-related ILD (RA-ILD) [8,9], scleroderma-related ILD [10] and IPF [11]. Reports also suggest that emphysema occurs in never-smoker patients with HP [[12], [13], [14]]. Our study therefore aimed to characterize the prevalence and functional and prognostic effects of emphysema and PPFE identified on CT imaging in a large population of patients with hypersensitivity pneumonitis.

## 2 Methods

### 2.1 Study population and clinical information

Patients with a multidisciplinary team diagnosis of HP, presenting between January 2007 to July 2014 with volumetric non-contrast CT examinations were identified. CT and pulmonary function protocols have been previously described [15]. Approval for this study of clinically indicated CT and pulmonary function data was obtained from the Institutional Ethics Committee of the Royal Brompton Hospital.

The pulmonary function indices examined included forced expiratory volume in the first second (FEV1), forced vital capacity (FVC), diffusing capacity for carbon monoxide (DLco), carbon monoxide transfer coefficient (Kco) and the composite physiological index (CPI) [16].

### 2.2 Visual CT analysis

Each CT scan was evaluated independently by two radiologists (AO, ALB) with 5 and 7 years thoracic imaging experience respectively, blinded to all clinical information. Both scorers were given 15 non-study cases to trial the scoring system and identify pre-existing biases. The scores were reviewed with a third scorer (JJ) prior to the scoring of the study cases.

Total interstitial lung disease extent was scored on a lobar basis (to the nearest 5%), in six lobes, with the lingula and upper lobe characterized separately in the left lung.

Three types of low attenuation lung, were quantified to the nearest 5% on a lobar basis and included expanded pulmonary lobules typical of HP, cysts and emphysema.

**Fig. 1:**
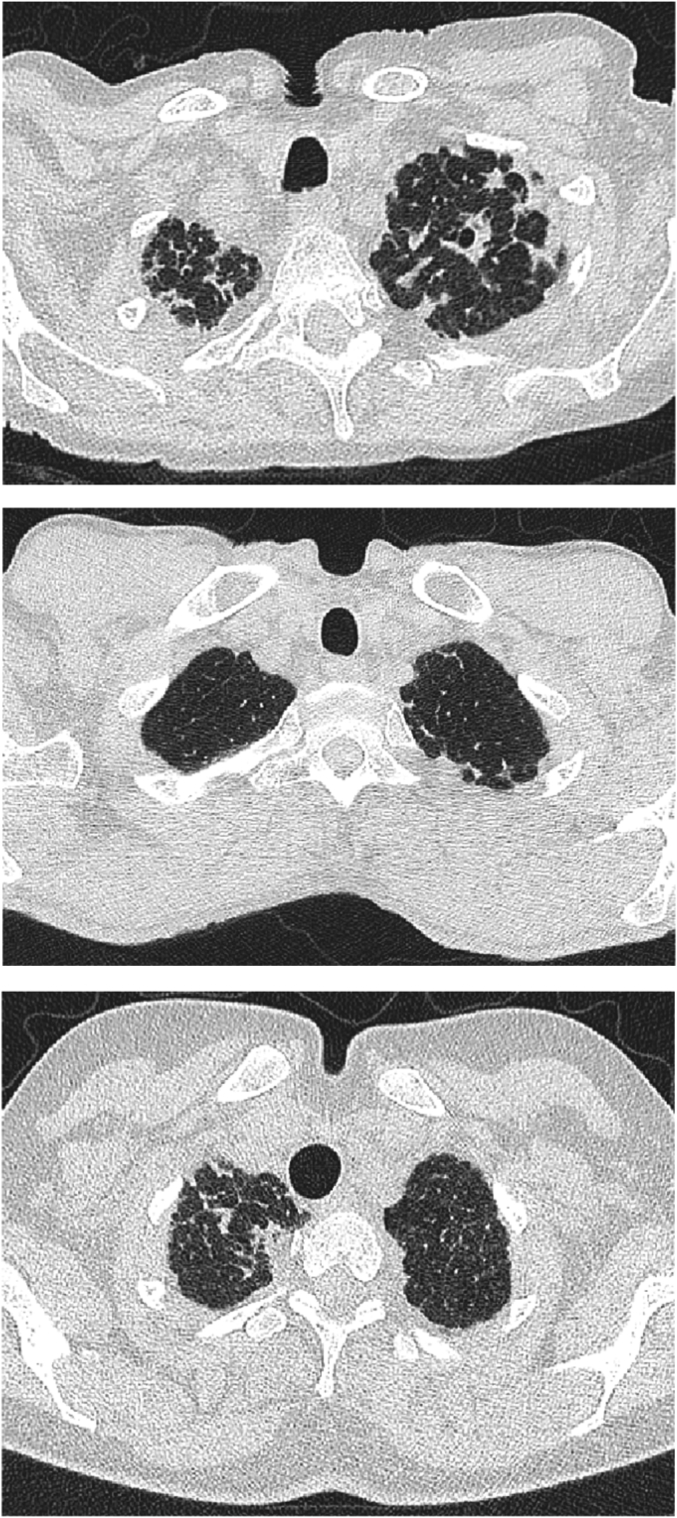
Axial CT images demonstrating a midline suprasternal depression which was only ever seen in hypersensitivity pneumonitis patients with pleuroparenchymal fibroelastosis (PPFE). The depression was invariably visible between the clavicles, and was often accompanied by a concavity of the skin in the midline of the back at the same level. Fig. 1a highlights dense pleural and parenchymal aggregations of fibrous tissue in a 71-year-old female never-smoker exposed to avian antigens. Fig. 1b demonstrates PPFE in the left upper lobe in a 59-year-old, male, never-smoker exposed to avian antigens and mould, with a diagnosis of hypersensitivity pneumonitis confirmed on surgical lung biopsy. Fig. 1c demonstrates PPFE in the right upper lobe in a 43-year-old, female, never-smoker with farmers lung, with a diagnosis of hypersensitivity pneumonitis confirmed on surgical lung biopsy.

The presence of pleuroparenchymal fibroelastosis was identified on a lobar basis (Fig. 1) using previously defined CT criteria [2]. PPFE was scored on a 4-point categorical scale as: 0 = absent, 1 = mild only affecting <10% of the pleural surface, 2 = moderate affecting 10–33% of the pleural surface, 3 = severe affecting >33% of the pleural surface. Arbitrated lobar PPFE scores were summed for each patient to create an overall 19-point (potential scores of 0–18) scale for total PPFE. Total PPFE scores were defined as follows: trivial = ≤2, marked≥3.

Bronchial wall thickening and dilatation, which have been linked to idiopathic PPFE [2] were evaluated on four-point categorical scales on a lobar basis, using scoring systems previously used to estimate bronchial wall thickening and bronchiectasis severity [[17], [18], [19]]: bronchial wall thickening: 0 = absent, 1 = mild, 2 = moderate, 3 = severe; bronchial dilatation: 0 = absent, 1 = dilatation not reaching CT criteria for bronchiectasis, 2 = mild dilatation of 1–1.5 times the size of the adjacent pulmonary artery, 3 = marked dilatation of more than 1.5 times the size of the adjacent pulmonary artery. The presence/absence of an obvious suprasternal depression was also noted (Fig. 1).

Finally the presence/absence of 4 CT features suggestive of non-PPFE related pleural thickening were noted: 1 = concentric smooth pleural thickening extending over 15% of the area of a hemithorax and lying distant to regions of PPFE, 2 = calcified pleural plaques consistent with asbestos exposure, 3 = fibrocalcific disease in the upper lobes compatible with old tuberculous infection [20], 4 = an apical cap, characterized as a density centered on the lung apex, and extending inferiorly (in the z-axis) as a continuous density for no more than 1 cm [21].

### 2.3 Consensus formulation

Differences in ILD, airway and low attenuation scores between observers, were visualized by plotting the spread of differences in each of the parenchymal pattern scores between observers. The graphs highlighted the variation in scores between observers for each individual CT feature, but also allowed appreciation of cohort-wide systematic biases held by each observer [15]. The most disparate 5% (2 SDs) of scores for each CT pattern were arbitrated by a third scorer (JJ). Disparity in any of the binary presence/absence scores was also arbitrated by the same third scorer (JJ).

As PPFE is a relatively new sign, arbitration was performed by an experienced radiologist with over 30 years of thoracic radiology practice (Professor David Hansell) to reduce the possibility of over-scoring of PPFE. Any disparities in the designation of the presence/absence of PPFE between scorers on a lobar basis, and any case in which the maximum lobar PPFE extent identified by both scorers was <10% (mild PPFE), was arbitrated by the experienced radiologist.

### 2.4 Statistical analysis

Data are given as means with standard deviations, or numbers of patients with percentages where appropriate. Interobserver variation for visual scores was assessed using the single determination standard deviation for continuous variables and the Kappa statistic for categorical variables. PPFE group differences for categorical variables was tested using the Kruskal Wallis test. Univariable and multivariable linear regression analyses were undertaken to investigate relationships between visual CT features and pulmonary function tests. Univariable and multivariable Cox regression analyses were undertaken to investigate determinants of mortality. In all study analyses, a p-value of <0.05 was considered significant. Linear regression models were formally tested for heteroscedasticity to confirm that the assumptions of parametric analysis had been satisfied. Statistical analyses were performed with STATA (version 12, StatCorp, College Station, TX, USA).

## 3 Results

### 3.1 Baseline data

The initial study population comprised 233 patients with a final multidisciplinary team diagnosis of HP based on a compatible clinical history and review of the following data: antigen exposure history (positive in 41% of patients), precipitating antibodies (identified in 35%), bronchoalveolar lavage findings (performed in 60%), CT imaging (100%) and histopathology from lung biopsy (40%). No treatment information could be obtained for 7 patients. In the remaining patients (n = 226), when considered on an intention to treat basis, 19/226 (8%) patients were observed and not given steroids or immunosuppression initially. 147/226 (65%) were initially treated with steroids alone. 21/266 (9%) patients were treated with immunosuppression alone. 39/226 (17%) were treated with both immunosuppression and steroids.

Demographic data, mean visual CT and pulmonary function tests results are shown in Table 1. 3/233 (1%) patients were censored due to incomplete follow-up, and the mean follow up time was 4.9 years. Never-smoker patients had a total lifetime tobacco exposure of less than 100 cigarettes [22]. Interobserver variation scores for ILD and low attenuation CT pattern extents are shown in Supplementary Table 1. Observer agreement for the presence of PPFE (k = 0.56) and for the presence of emphysema (k = 0.44) was moderate (Supplementary Table 2). Observer agreement improved with more extensive emphysema extents (Supplementary Table 2).

**Table 1:** Patient age, gender and mean and standard deviations of pulmonary function indices and visually scored CT parameters in all patients with hypersensitivity pneumonitis (Column 1), and subdivided according to patients with none/trivial PPFE and marked PPFE. Data represent mean values with standard deviations. FEV1 = forced expiratory volume in the first second, FVC = forced vital capacity, DLco = diffusing capacity for carbon monoxide, Kco = carbon monoxide transfer coefficient, CPI = composite physiological index, ILD = interstitial lung disease, PPFE = pleuroparenchymal fibroelastosis.* = p < 0.001, ˆ = p < 0.01,^#^=<0.05.

Variable (n = 233 unless stated)	Entire Cohort	None/trivial PPFE (n = 180 unless stated)	Marked PPFE (n = 53 unless stated)
Median Age (range)	62 (32–53)	63 (35–83)	56 (32–76)ˆ
Male/female	93/140	78/102	15/38^#^
Identifiable antigen (%)	41	45	49
Survival (alive/dead)	140/93	112/68	28/25
Never smokers/ever-smokers (n = 230)	142/88	102/77	40/11ˆ
Pack years for smokers alone (n = 85)	17.9 ± 15.4	19.1 ± 15.7 (76)	7.6 ± 6.7 [9]
FEV1% predicted (n = 220)	68.2 ± 21.4	71.6 ± 21.2 (170)	56.3 ± 17.6 (50)*
FVC % predicted (n = 222)	69.8 ± 24.3	73.4 ± 24.5 (172)	57.5 ± 19.1 (50)*
FEV1/FVC % predicted (n = 220)	80.5 ± 9.2	79.9 ± 9.3 (170)	82.3 ± 8.6 (50)
DLco % predicted (n = 211)	41.8 ± 17.4	43.7 ± 18.1 (166)	34.9 ± 12.6 (45)*
Kco % predicted (n = 211)	70.6 ± 18.6	71.4 ± 19.2 (166)	67.7 ± 16.2 (45)
CPI (n = 208)	49.5 ± 16.4	47.7 ± 17.0 (164)	56.5 ± 11.8 (44)ˆ
CT scores (%)			
Total ILD extent	47.6 ± 24.0	46.9 ± 25.2	49.7 ± 19.8
Mosaic attenuation	5.8 ± 7.4	5.8 ± 7.5	5.8 ± 7.0
Emphysema	2.2 ± 6.7	2.4 ± 7.4	1.5 ± 3.3
Cysts	0.5 ± 1.6	0.4 ± 1.4	0.7 ± 2.2
PPFE/airway scores (maximum score = 19)			
PPFE extent (all patients)	1.6 ± 2.7	0.4 ± 0.7	5.5 ± 3.0*
PPFE extent (patients with PPFE only)	3.9 ± 3.0	3.9 ± 3.0	3.9 ± 3.0
Bronchial dilatation	0.6 ± 1.8	0.6 ± 1.7	0.8 ± 2.0
Bronchial wall thickening	5.2 ± 4.9	5.5 ± 4.8	4.2 ± 5.0

### 3.2 Morphological/functional relations of PPFE

PPFE was identified in 93/233 (40%) patients and was trivial in 40/233 (17%) and marked in 53/233 (23%). PPFE was never seen in any of the middle or lower lobes without being present in both upper lobes. In 18/93 (19%) patients, PPFE was identified in the lower lobes without being present in either middle lobe. Only 20 patients had surgical lung biopsies (middle and lower lobes) performed or reviewed after 2012, when PPFE was becoming better recognised as a clinical entity. Of these 20 patients, only 4 demonstrated marked PPFE on CT. Intra-alveolar predominant fibrosis was seen in 3/4 of the cases with marked PPFE, with two of the cases demonstrating increased elastosis, in spite of the fact that the biopsies obtained were distant to the location of the PPFE.

Regarding accessory PPFE features, a suprasternal depression was seen in 23/233 (10%) study patients. 3/40 (8%) patients with trivial PPFE and 20/53 (38%) patients with marked PPFE had a suprasternal depression on CT (p = 0.001). An apical cap was identified in 90/233 (39%) patients; which included 24/140 (17%) patients with no PPFE, 24/40 (60%) patients with trivial PPFE and 42/53 (79%) patients with marked PPFE (p < 0.0001). Smooth pleural thickening distinct from regions of PPFE (3/233 [1%] patients), granulomas suggestive of previous tuberculous infection (1/233 [<1%] patients) and pleural plaques suggestive of previous asbestos exposure (6/233 [3%] patients) were rarely seen in the study population.

No functional difference (FVC, DLco or CPI) was identified between patients with no PPFE and patients with trivial PPFE, after adjusting for covariates (patient age, gender, ILD and emphysema extent and smoking status [never versus ever]). Consequently, in all further PPFE analyses, patients with trivial and no PPFE were combined, and examined against patients with marked PPFE.

On univariate logistic regression, a positive smoking history was negatively associated with marked PPFE (OR = 0.36, 95%CI 0.18–0.76, p = 0.007). Marked PPFE was independently associated with a reduction in FVC and DLco following adjustment for covariates (patient age, gender, ILD extent, smoking status [never versus ever] and emphysema extent)[Table 2], with results maintained when examined selectively in never-smoker HP patients (Supplementary Table 3).

**Table 2:** Relationships between pulmonary function tests (FEV1/FVC ratio, FVC and DLco) and interstitial lung disease (ILD) and emphysema extents (measured as percentages), smoking status (never versus ever) and PPFE (marked versus none/trivial) in patients with hypersensitivity pneumonitis. All models were adjusted for patient age and gender. FEV1 = forced expiratory volume in the first second, FVC = forced vital capacity, DLco = diffusing capacity for carbon monoxide, PPFE = pleuroparenchymal fibroelastosis.

Dependent variable	CT variable	Beta Coefficient	95% Confidence Interval	P value	Model R value
**FEV1/FVC**	ILD extent	0.09	0.05, 0.14	0.0001	0.47
	Smoking status	−1.68	−4.04, 0.68	0.18	
	Emphysema extent	−0.45	−0.62, −0.28	<0.0001	
	Marked PPFE	1.02	−1.79, 3.83	0.48	
**FVC**	ILD extent	−0.50	−0.61, −0.39	<0.0001	0.60
	Smoking status	5.47	−0.13, 11.07	0.06	
	Emphysema extent	0.44	0.03, 0.84	0.03	
	Marked PPFE	−12.67	−19.36, −5.98	0.0002	
**DLco**	ILD extent	−0.39	−0.47, −0.31	<0.0001	0.62
	Smoking status	3.35	−0.72, 7.41	0.11	
	Emphysema extent	−0.37	−0.66, −0.08	0.01	
	Marked PPFE	−7.80	−12.75, −2.86	0.002	

### 3.3 Functional relationships of emphysema

**Fig. 2:**
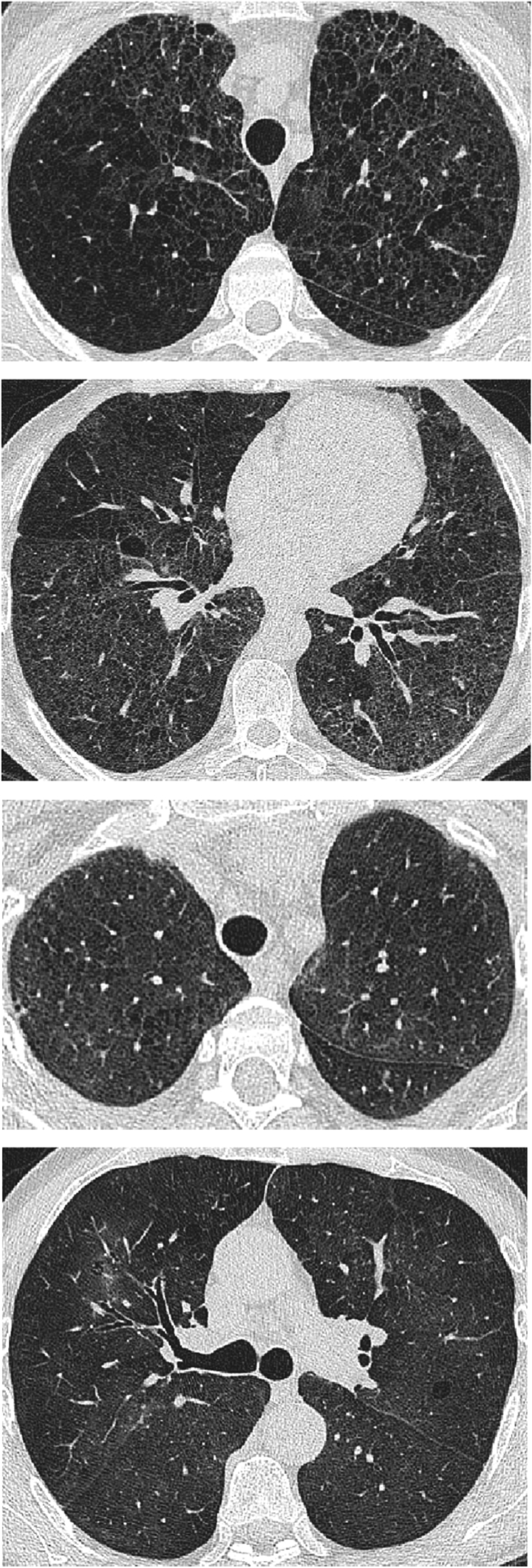
Axial CT image of the lungs demonstrating emphysema in never-smoker patients with hypersensitivity pneumonitis. Fig. 2a and b demonstrate the upper and lower lobes respectively of a 61-year-old male never-smoker who kept birds and had avian and micropolysporia antibodies. Lucencies in keeping with emphysema are visible throughout both lungs and demonstrate an upper lobe predominance. Fig. 2c demonstrates the upper lobes of a 51-year-old female never-smoker with an unknown antigen exposure. Centrilobular lucencies are visible in both upper lobes, but predominate in the right lung. Fig. 2d demonstrates the lung midzones in a 76-year-old male never-smoker, again with no known antigen exposure. Centrilobular emphysematous foci are seen bilaterally, several of which contain a central vessel, with one lucency in the right upper lobe surrounded by inflammatory ground glass density.

Emphysema was seen in 70/233 (30%) HP patients, of which 37/88 (42%) patients were smokers and 33/142 (23%) were never-smokers (Fig. 2). 11/33 (33%) never-smoker patients with HP had >5% emphysema on CT. When examining all study patients with emphysema, the mean emphysema extent was 7.2 ± 10.6%, with no significant difference seen in emphysema extent between smokers and never-smokers (Supplementary Table 4). Emphysema in ex-smokers and never-smokers was mostly a combination of centrilobular and paraseptal emphysema with a heavily upper lobe predominant distribution. No statistical difference was seen in any low attenuation CT pattern extent between smokers and never-smokers (Supplementary Table 4).

Patients with emphysema demonstrated preservation of lung volumes relative to patients without emphysema and similar mean DLco values to patients without emphysema despite having less extensive ILD (Supplementary Table 5). Multivariable linear regression analyses were performed to exclude a confounding effect on functional indices from covariates (age, gender, baseline ILD extent, smoking status and PPFE severity [marked versus none/trivial]), and across all study patients, emphysema extent demonstrated a CPFE functional phenotype with preservation of lung volumes and a disproportionate reduction in DLco (Table 2). In never-smokers, when using the same multivariable models, emphysema extent was associated with obstructive functional indices, and a disproportionate reduction in DLco, though preservation of lung volumes did not reach statistical significance (Supplementary Table 3). When functional relationships were re-examined in patients that had received treatment (n = 207), 23% of the study population with >5% emphysema were excluded (as they were either managed by observation or did not have treatment information available). As a result, whilst functional trends were maintained, underpowering resulted in the independent FVC and DLco relationships with emphysema extent not retaining significance.

### 3.4 PPFE and emphysema mortality associations

On univariable Cox mortality analysis, emphysema presence, emphysema extent and marked PPFE did not significantly predict mortality. In multivariable models adjusted for patient age, gender, smoking status, ILD extent and PPFE (marked versus none/trivial), marked PPFE independently predicted mortality (HR = 1.97, 95%CI 1.16–3.33, p = 0.01) unlike emphysema extent (Table 3).

**Table 3:** Multivariable Cox regression models demonstrating mortality in HP patients adjusted for patient age, gender, ILD and emphysema extents, smoking status and pleuroparenchymal fibroelastosis (PPFE) severity. The second model also examined emphysema presence (representing the combined pulmonary fibrosis and emphysema phenotype). ILD=Interstitial lung disease.

Variable	Hazard ratio Adj(unadj)	95.0% Confidence Interval		P Value
		Lower	Upper	
Age (years)	1·06 (1.05)	1·03	1·08	<0.0001
Male Gender	1·19 (1.17)	0.75	1.88	0·46
Ever smoker	1.31 (1.31)	0.84	2.04	0.23
ILD extent (%)	1.02 (1.02)	1.01	1.03	<0.0001
Emphysema extent (%)	1.02 (1.00)	0.98	1.05	0.33
PPFE (marked vs none/trivial)	1.94 (1.25)	1.15	3.27	0.01
Age (years)	1·06 (1.05)	1·03	1·08	<0.0001
Male Gender	1·25 (1.17)	0.79	1.97	0·35
Ever smoker	1.38 (1.31)	0.88	2.15	0.16
Summed ILD and emphysema extent (%)	1.02 (1.02)	1.01	1.03	<0.0001
PPFE (marked vs none/trivial)	2.04 (1.25)	1.20	3.47	0.008
Emphysema presence	0.73 (0.90)	0.45	1.18	0.19

When emphysema presence, representing the CPFE phenotype was inserted into a Cox model containing patient age, gender, smoking status, summed ILD and emphysema extent (representing the combined morphological extent of parenchymal damage) and PPFE (marked versus none/trivial), emphysema presence was not significantly associated with outcome (Table 3).

All multivariate Cox model findings were maintained when re-examined with adjustment for antigen exposure (identified antigen vs no identified antigen) and in selective subanalyses of patients that received treatment (steroids and/or immunosuppression – classified on an intention to treat basis). All multivariate Cox model findings were also maintained when PPFE severity and smoking status were omitted from the models.

## 4 Discussion

Our study is the first comprehensive examination of the prevalence and impact of PPFE in HP patients. Marked PPFE was identified in 23% of HP patients and was independently associated with a reduced FVC and DLco, and, importantly, was independently predictive of mortality. HP patients with emphysema demonstrated the typical CPFE profile of artificially preserved lung volumes with a disproportionate reduction in DLco, and emphysema was identified in 23% of never smokers. We have also demonstrated that HP patients with CPFE do not have a worsened outcome compared to HP patients without emphysema, beyond that explained by CT extents of ILD and emphysema, a finding which parallels outcome observations with regard to CPFE in patients with IPF [11].

Previous studies of PPFE have relied on histological correlation of imaging findings [2,[4], [5], [6], [23],^23^], and as a result have examined series with limited patients numbers. Yet the CT appearances of PPFE are now well described, having been included in the consensus statement for the multidisciplinary classification of the idiopathic interstitial pneumonias [3]. Consequently, as per the study of Enomoto et al. [24], which examined CT appearances of patients with idiopathic PPFE, it is now possible to interrogate large CT populations to determine the prevalence and functional and prognostic effects of PPFE.

The well documented upper lobe predilection of PPFE [1,2,23,25] was confirmed in the current study, as PPFE was never found in a middle or lower lobe without being evident in an upper lobe. Every patient in the current series that demonstrated a suprasternal depression was found to have trivial or marked PPFE, indicating the strong association of the CT sign with PPFE. A similar linkage has been previously noted between PPFE and a narrowed antero-posterior thoracic diameter [4,26]. The presence of an apical cap was significantly more common when PPFE was marked, but demonstrated no relation to sequelae of previous tuberculous exposure.

A positive smoking history was found to protect against the development of PPFE in the current study, confirming previous observations demonstrating idiopathic PPFE to be more common in non-smokers [1,2,7,[27], [28], [29]]. The reasons for the apparent protective effect of smoking in PPFE are unclear. However, as PPFE can be conceptualized as an expression of immune dysregulation, it is possible that loss of vessels in the upper zone associated with smoking-related lung damage may limit the development of PPFE on the visceral pleura.

The low baseline FVC identified in the current study is in line with previous observations in idiopathic PPFE [23,24,29], however the mean study DLco was lower than previous idiopathic PPFE reports [23,24] which probably reflects the co-existent fibrosis in our HP population. Our observation that marked PPFE independently links to mortality also confirms observations in patients with idiopathic PPFE who progressed with rapid declines in FVC [5,23,29].

With regard to our detailed emphysema evaluation, emphysema across the entire study population was primarily centrilobular and paraseptal in nature with an upper lobe predominant distribution. The emphysema also demonstrated obstructive functional indices with a CPFE functional profile. The findings together suggest that parenchyma labelled as emphysema is unlikely to have reflected misclassified honeycombing. The aetiology of the emphysema identified in 23% of never smokers cannot be verified but may reflect autoimmune [30] or inflammatory parenchymal damage in a population where immune dysregulation is well recognised [31]. Proportions of never-smokers with emphysema are also comparable to previous reports in IPF [11] and RA-ILD [9]. The observation that HP patients with CPFE have no worsened outcome that HP patients without emphysema once baseline extents of ILD and emphysema have been considered also corroborates similar findings in IPF [11] and RA-ILD [9].

There were limitations to the study. Firstly, the lack of comprehensive histopathological sampling to confirm the presence of PPFE might be seen as a major omission. However, given the detailed CT descriptions of PPFE over the past 15 years, we believe that a PPFE diagnosis can be made with confidence without histopathological corroboration. Furthermore, we aimed to quantify PPFE severity across the entirety of the lungs, which would have been unachievable had we been reliant on biopsy-based sampling. We also did not have histopathological proof of an HP diagnosis. Yet in an era where surgical biopsies are rarely performed in patients with fibrosing lung disease, and guidelines for the diagnosis of HP are awaited, reliance on a multidisciplinary team diagnosis remains the gold standard, to which we adhered.

A further limitation related to the subanalysis of the independent functional effects of emphysema in HP patients receiving treatment. Whilst trends of preserved lung volumes and DLco reduction were maintained, the analyses were underpowered and did not retain significance. Corroboration that the functional trends in CPFE patients identified in our study are independent of patient treatment, would best be achieved through analyses of large multicentered patient populations. Lastly, the kappa values for interobserver agreement for the presence of emphysema were moderate, but improved in patients with more extensive emphysema extents. Arbitration of discrepant emphysema scores primarily focused on patients with minor emphysema extents, and the adequacy of the consensus findings are reflected in the good functional correlations of parenchyma classified as emphysematous. Whilst better agreement for the presence of emphysema between observers would be preferable, the findings in the current study are in line with previous reports [9,11] and reflect the challenges inherent in characterizing CT patterns, recognised throughout the ILD literature. Regarding observer agreement for PPFE in the literature however, there is limited information for patient cohorts of a comparable size to the current study.

In conclusion, our study has demonstrated that in patients with HP, marked PPFE, which is associated with reduced lung volumes and gas transfer, is not uncommon and had a prevalence of 23% in our study cohort. We have shown that HP patients with emphysema demonstrate the characteristic lung function profile of CPFE, and in our study cohort, 23% of never-smokers with HP demonstrated emphysema on CT imaging. We demonstrate that CPFE in HP is not associated with a worsened outcome, when compared to HP patients without emphysema, once CT extents of emphysema and ILD have been considered. However, marked PPFE does independently predict mortality in HP.

## Ethics committee approval

Approval for this study of clinically indicated CT and pulmonary function data was obtained from the Institutional Ethics Committee of the Royal Brompton Hospital and informed patient consent was not required.

## Conflicts of interest

Dr. Jacob reports personal fees from Boehringer Ingelheim outside the current work.

Dr. Renzoni reports personal fees from Roche, Boehringer Ingelheim, and Takeda, outside the submitted work.

Dr. Devaraj reports personal fees from Roche and Boehringer Ingelheim, outside the submitted work.

Prof Wells reports personal fees from Intermune, Boehringer Ingelheim, Gilead, MSD, Roche, Bayer, and Chiesi outside the submitted work.

## Authors contributions

JJ, MK, AO, ALB, AUW, CM, AL, AD SD, ER were involved in either the acquisition, or analysis or interpretation of data for the study.

JJ and AUW were also involved in the conception and design of the study.

All authors revised the work for important intellectual content and gave final approval for the version to be published. All authors agree to be accountable for the all aspects of the work in ensuring that questions related to the accuracy or integrity of any part of the work are appropriately investigated and resolved.

JJ had full access to all the data in the study and takes responsibility for the integrity of the data and the accuracy of the data analysis, including and especially any adverse effects. JJ assumes full responsibility for the integrity of the submission as a whole, from inception to published article.

## Appendix A Supplementary data

eAppendix eAppendix
