# Supplementary material for: Functional associations of pleuroparenchymal fibroelastosis and emphysema with hypersensitivity pneumonitis
Source: Respir Med. 2018 May;138:95–101. doi: 10.1016/j.rmed.2018.03.031 (PMC5948318; doi:10.1016/j.rmed.2018.03.031)
Supplement: eAppendix [file mmc1.docx]

**eAppendix**

| **Visual CT Variable** | **Single determination standard deviation** |
| --- | --- |
| **Total interstitial lung disease** | 11.3 |
| **Mosaicism** | 4.4 |
| **Emphysema** | 2.9 |
| **Cysts** | 3.8 |

eTable 1. Variation in interstitial and low attenuation pattern scores between the two scorers evaluated using the single determination standard deviation.

| **Visual CT Variable** | **Kappa Statistic** |
| --- | --- |
| **PPFE presence** | 0.56 |
| **Marked PPFE presence** | 0.52 |
| **Emphysema presence** | 0.44 |
| **Emphysema >5% presence** | 0.62 |
| **Suprasternal depression** | 0.57 |
| **Apical cap presence** | 0.59 |

eTable 2. Variation in categorical visual CT scores between the two scorers evaluated using the Kappa statistic. PPFE=pleuroparenchymal fibroelastosis.


| **Dependent variable** | **CT variable** | **Beta**  **Coefficient** | **95% Confidence Interval** | **P value** | **Model R value** |
| --- | --- | --- | --- | --- | --- |
| **FEV1/FVC** | ILD extent | 0.09 | 0.03, 0.15 | 0.004 | 0.40 |
|  | Emphysema extent | -0.35 | -0.55, -0.15 | 0.001 |  |
|  | Marked PPFE | 1.54 | -1.82, 4.91 | 0.37 |  |
| **FVC** | ILD extent | -0.51 | -0.66, -0.37 | <0.0001 | 0.57 |
|  | Emphysema extent | 0.31 | -0.17, 0.78 | 0.20 |  |
|  | Marked PPFE | -11.31 | -19.24, -3.38 | 0.006 |  |
| **DLco** | ILD extent | -0.43 | -0.53, -0.34 | <0.0001 | 0.67 |
|  | Emphysema extent | -0.49 | -0.80, -0.18 | 0.002 |  |
|  | Marked PPFE | -7.18 | -12.42, -1.94 | 0.008 |  |

eTable 3. Relationships between pulmonary function tests (FEV1/FVC ratio, FVC and DLco) and ILD and emphysema extent (expressed as a percentage) and PPFE (marked versus none/trivial) in never-smoker patients with hypersensitivity pneumonitis. All models were adjusted for patient age and gender. FEV1=forced expiratory volume in one second, FVC=forced vital capacity, DLco=diffusing capacity for carbon monoxide, ILD=interstitial lung disease.

| **Low attenuation CT subtypes**  **(%)** | **Smokers**  **(n=86)** | **Never smokers**  **(n=144)** | **P value** |
| --- | --- | --- | --- |
| **Mosaicism** | 5.0±7.9 | 6.2±7.1 | 0.24 |
| **Emphysema** | 2.9±5.6 | 1.8±7.3 | 0.20 |
| **Cysts** | 0.4±1.1 | 0.5±1.9 | 0.83 |
| **Emphysema alone*** | 6.9±6.9 | 7.6±13.6 | 0.79 |

eTable 4. Mean percentage extents of three low attenuation CT patterns in smokers and never-smokers with hypersensitivity pneumonitis. A subanalysis (*) was performed selectively in patients with emphysema. Group differences were evaluated with the T-test.


| **Dependent variable** | **No Emphysema**  **(cohort size)** | **Emphysema**  **(cohort size)** | **P value** |
| --- | --- | --- | --- |
| **FEV1/FVC** | 82.2 ± 7.7 (157) | 76.2 ± 11.1 (63) | <0.003 |
| **FVC** | 67.6 ± 23.8 (157) | 75.2 ± 24.8 (65) | 0.04 |
| **DLco** | 41.8 ± 17.5 (148) | 41.9 ± 17.4 (63) | 0.95 |
| **VILD %()** | 48.8 ± 23.7 (163) | 44.8 ± 24.9 (70) | 0.26 |
| **VILDemph (%)** | 48.8 ± 23.7 (163) | 52.0 ± 25.2 (70) | 0.36 |

eTable 5. Differences in pulmonary function tests (% predicted FEV1/FVC ratio, FVC and DLco) and CT pattern extents (VILD=visual interstitial lung disease extent; VILDemph=sum of visual ILD and emphysema extents representing the combined morphological extent of disease on CT) in hypersensitivity pneumonitis patients with and without emphysema. FEV1=forced expiratory volume in one second, FVC=forced vital capacity, DLco=diffusing capacity for carbon monoxide.
